# Bacteriophage-Resistant Mutant of *Enterococcus faecalis* Is Impaired in Biofilm Formation

**DOI:** 10.3389/fmicb.2022.913023

**Published:** 2022-06-09

**Authors:** Jiazhen Liu, Yanpeng Zhu, Yang Li, Yuwen Lu, Kun Xiong, Qiu Zhong, Jing Wang

**Affiliations:** ^1^Department of Clinical Laboratory Medicine, Daping Hospital, Army Medical University, Chongqing, China; ^2^Department of Microbiology, Army Medical University, Chongqing, China; ^3^Department of Oral and Maxillofacial Surgery, Southwest Hospital, Army Medical University, Chongqing, China; ^4^Medical Center of Trauma and War Injury, Daping Hospital, Army Medical University, Chongqing, China; ^5^Department of Frigidzone Medicine, College of High Altitude Military Medicine, Army Medical University, Chongqing, China

**Keywords:** bacteriophage, *Enterococcus faecalis*, phage resistance, phage receptor, capsular polysaccharide, biofilm

## Abstract

*Enterococcus faecalis* is a common gram-positive non-spore-forming bacterium in nature and is found in the upper respiratory tract, intestine, and mouth of healthy people. *E. faecalis* is also one of the common pathogens causing nosocomial infections and is resistant to several antibiotics commonly used in practice. Thus, treating drug-resistant *E. faecalis* with antibiotics is challenging, and new approaches are needed. In this study, we isolated a bacteriophage named EFap02 that targets *E. faecalis* strain EFa02 from sewage at Southwest Hospital. Phage EFap02 belongs to the *Siphoviridae* family with a long tail of approximately 210 nm, and EFap02 can tolerate a strong acid and alkali environment and high temperature. Its receptor was identified as the capsular polysaccharide. Phage-resistant mutants had loss-of-function mutations in glycosyltransferase (*gtr2*), which is responsible for capsular polysaccharide biosynthesis, and this caused the loss of capsular polysaccharide and interruption of phage adsorption. Although phage-resistant mutants against EFap02 can be selected, such mutants are impaired in biofilm formation due to the loss of capsular polysaccharide, which compromises its virulence. Therefore, this study provided a detailed description of the *E. faecalis* EFap02 phage with the potential for treating *E. faecalis* infection.

## Introduction

*Enterococcus faecalis* is a common opportunistic pathogen that causes blood and urinary tract infections. It is one of the primary pathogens that infect root canals (Khalifa et al., 2016). *E. faecalis* is intrinsically resistant to several commonly used antibiotics, such as cephalosporin and aminoglycoside (García-Solache and Rice, 2019). *Vancomycin-resistant Enterococcus* (VRE) is resistant to vancomycin (Miller et al., 2016). With the emergence of multidrug-resistant strains of *E. faecalis* and its ability to form biofilms (Ch'ng et al., 2019), *E. faecalis* infection has caused great concern in practice (Fiore et al., 2019), and there is an urgent need to find new treatments.

In recent years, phage therapy has renewed interest as a promising alternative to antibiotics (Khalifa et al., 2016). Phage therapy has successfully treated patients with multidrug-resistant bacterial infections. In Belgium and France, a randomized, controlled, double-blind phase I/II trial (PhagoBurn) used a cocktail of 12 natural lytic phages to treat burn wounds infected with *Pseudomonas aeruginosa*. Compared to standard care, the bacterial load was reduced in the phage cocktail treatment group, indicating that phage therapy is promising, although the quality of the phage product needs improvement (Jault et al., 2019). Furthermore, in China, a 63-year-old woman who developed a recurrent urinary tract infection with extensively drug-resistant *Klebsiella pneumoniae* was cured by antibiotic and phage synergism (Bao et al., 2020). Only one human study described the treatment of chronic prostatitis associated with *E. faecalis* by phage therapy in 2007. Three patients who suffered chronic *E. faecalis* infection were not cured with antibiotics, autovaccines, and laser biostimulation. They were then treated with phage therapy twice daily for 1 month. The pathogen was eradicated, and clinical symptoms were relieved without recurrence (Letkiewicz et al., 2009).

In this study, we successfully isolated a lytic phage, EFap02, from the sewage of Southwest Hospital in China. Phage EFap02 belongs to the *Siphoviridae* family and has a potent lytic effect against *E. faecalis.* The capsular polysaccharide was identified as the EFap02 receptor. The phage-resistant mutant had loss-of-function mutations in glycosyltransferase Group 2 (*gtr2*), which is responsible for capsular polysaccharide biosynthesis. The mutations caused the loss of capsular polysaccharide and interruption of phage adsorption. The loss of capsular polysaccharide significantly decreased the biofilm formation capability compromising the EPap02 virulence. Overall, this study suggested that EFap02 could be a promising candidate for *E. faecalis* phage therapy.

## Materials and Methods

### Strains and Cultural Conditions

The bacterial strains and phages are listed in Table 1. The EFa02 strain was isolated from the Daping Hospital Department of Clinical Laboratory Medicine. *E. faecalis* was cultured in brain heart infusion (BHI) medium at 37°C. When necessary, erythromycin (20 μg/ml) was added to the medium. The stock cultures were stored in medium supplemented with 20% glycerol at −80°C.

**Table 1:** Bacterial strains and phages used in this study.

**Strain or phage**	**Description**	**Source**
EFa02	Wild type *Enterococcus faecalis* strain	This study
EFa02R EFa02R*::gtr2*	Phage-resistant mutant EFa02R complemented with *gtr2*	This study This study
EFap02	Phage targets EFa02	This study

### Isolation of the Phage That Infects *Enterococcus faecalis*

Isolation of bacteriophages was performed as previously described (Shen et al., 2018). Sewage (10 ml) from the Southwest Hospital was centrifuged at 10,000 × g for 10 min. The supernatant was filtered with a 0.45 μm aseptic filter, and 2 ml of the filtered supernatant was added to a 50 ml clean centrifuge tube. The logarithmic phase host bacteria EFa02 was added and then cultured in a 37°C shaking incubator overnight. The mixture was centrifuged at 21,000 × g for 1 min, and the supernatant was filtered using a 0.22 μm aseptic filter. Then, 10 μl supernatant was mixed with 100 μl host bacteria EFa02 in a 15 ml centrifuge tube. BHI soft agar (5 ml) was added, and the content was poured onto the surface of agar plates. The plates were incubated at 37°C overnight, and the plaques were observed on the top agar.

### Electronic Microscope

Phage morphology was observed by transmission electron microscopy (TEM; HT7700, Hitachi, Japan; Lee et al., 2019). The filtered phage lysate was dropped onto the prepared Formvar/carbon-coated copper grid and incubated for 1 min. The grid was negatively stained with 2% uranium acetate for 10 min and then observed using TEM at an acceleration voltage of 80 kV. Phages are classified according to the guidelines of the International Committee on the Taxonomy of Viruses (ICTV; Lefkowitz et al., 2018).

### Determination of the Optimal Multiplicity of Infection

The optimal multiplicity of infection (MOI) is the ratio of bacteriophages to bacteria at the time of infection. The optimal MOI is the number of infections when the phage can achieve the best growth state (Lee et al., 2019). According to different MOIs, the phage (2 ml) and host bacteria (2 ml) were mixed. BHI medium was added to the mixture to a final volume of 10 ml. The mixture was incubated at 37°C with shaking at 220 rpm for 6 h. The 1 ml mixture was centrifuged at 12,000 × g for 1 min and filtered with a 0.45 μm filter. The phage titer was determined by the double-layer agar (DLA) method (Lee et al., 2019). The MOI with the highest phage titer was the optimal MOI. Three biological repeats were performed.

### One-Step Growth Curve

As previously described, one-step phage growth was performed (Lu et al., 2013). EFa02 was cultivated to the early logarithmic stage (OD_600_ = 0.5). The phage was mixed with EFa02 (10^8^ CFU/ml) at an MOI of 0.01 and incubated at 37°C with shaking at 220 rpm. Samples were taken at time points 0, 10, 20, 30, 40, 50, 60, and 90 min. The phage titer was measured using the DLA method. Three biological replicates were performed.

### pH and Thermal Stability of EFap02

The BHI medium was adjusted with HCl or NaOH to pH 2.0–14.0. Then, 990 μl of BHI medium was mixed with 10 μl phage stock solution (3 × 10^8^ PFU/ml). After incubation at 37°C for 60 min, the phage titer was determined by the DLA method.

BHI medium (900 μl) was mixed with 100 μl phage stock solution (9 × 10^9^ PFU/ml) and treated at 4, 25, 37, 50, 60, and 70°C. After 60 min, the samples were removed and cooled to room temperature. The phage titer was determined by the DLA method (Ding et al., 2020).

### Sensitivity of EFap02 to Chloroform

Phage EFap02 (10^10^ PFU/ml) was mixed with chloroform at ratios of 0, 10, 25, 50, 75, and 95%. The mixtures were incubated at 37°C with shaking at 220 rpm for 60 min. And the phage titer was determined using the DLA method (Oduor et al., 2020).

### Selection of Phage-Resistant Mutants

Isolating phage-resistant mutants was performed as described previously (Yang et al., 2020). Briefly, 10 μl of *E. faecalis* culture (OD_600_ = 0.2) was added to EFap02 (10^9^ PFU), and the mixture was placed on BHI agar and incubated at 37°C for 24 h. Then, the phage resistance of the colonies was validated by the DLA assay.

### Phage Genome Extraction, Sequencing, and Analysis

The extraction of the phage genome was performed as previously described with a slight modification (Yuan et al., 2020). To remove contaminated DNA and RNA from the phage stocks, DNase I and RNase A were added to a final concentration of 1 μg/ml, and the phage stocks were incubated at 37°C for 60 min. EDTA (0.5 mol/l) was added to a final concentration of 0.02 mol/l. Proteinase K (20 g/l) and 10% SDS were added (final concentration 0.5%). The mixture was treated at 56°C for 60 min. An equal volume of balanced phenol (pH = 8.0) was added to extract nucleic acids, and then, the mixture was centrifuged at 10,000 × g for 5 min. The upper aqueous phase was transferred to a new 1.5 ml centrifuge tube. An equal volume of chloroform was added. The tube was gently mixed and centrifuged at 10,000 × g for 10 min. The supernatant was transferred to a new centrifuge tube, mixed with a 0.6 volume of isoamyl alcohol, and placed at −20°C for 1 h. The mixture was centrifuged at 12,000 × g for 20 min to collect the DNA pellet. The DNA pellet was washed with 75% ice-cold ethanol and centrifuged at 12,000 × g for 20 min. Finally, the pellet was dried at room temperature, dissolved in 50 μl of water, and stored at −20°C for future use.

The Bacterial Genomic DNA Kit (Tiangen-DP302) was used for DNA extraction. The phage genome was sequenced using the Illumina HiSeq 2,500 platform (~1 Gbp/sample) and assembled with Newbler (Version 2.9) under default parameters. The characteristics of the phage genes were predicted by RAST^1^ (Aziz et al., 2008) and FgeneSV.^2^ The homologous DNA sequences and proteins were searched and analyzed using BlastN and BlastP in the BLAST program^3^ (Altschul et al., 1997). Visualization of the phage circular genome was performed using the CGView server database (Stothard and Wishart, 2005). Phage virulence factors were analyzed by searching a virulence database (Underwood et al., 2005). A comparative analysis of the EFa02 genomes and phage-resistant mutants was performed by Breseq (Barrick et al., 2014). Analysis of phage DNA termini was performed using PhageTerm^4^ (Garneau et al., 2017), and the phylogenetic tree was constructed and displayed by MEGA 7.0^5^ with the neighbor-joining method (Tamura et al., 2013).

### Bacterial Growth Curve and Construction of *Enterococcus faecalis Strains*

EFa02 and EFap02 were cocultured for 72 h to explore the effect of phage on bacterial growth. The OD_600_ of the culture medium was measured at given time points. Three biological replicates were performed. The *gtr2* gene was amplified by PCR using the primers listed in Table 2 to complement *gtr2* in EFa02R. The PCR product was ligated into the plasmid pGM23 by Gibson assembly to generate pGM-*gtr2,* and pGM-*gtr2* was electroporated into EFa02R, and the strain was named EFa02R::*gtr*2.

**Table 2:** Primers used in this study.

**Primer use or name**	**Sequence (5′–3′)**
Complementing with *gtr2*	
*pMG44-F*	TTCAAAATTCCTCCGAAT
*pMG44-R*	CCGGCGCTACGATATT
*pMG44-02R-F*	AAAATATTCGGAGGAATTTTGAAATGCCCAAAATTAGTATTATTGTTCC
*pMG44-02R-R*	ATATCGTAGCGCCGGTTAACTATTCTTTTTATTATTTGCTTCTTGTAATTTAGG

### Phage Adsorption Assay and Efficiency of Plating Assay

Phage adsorption tests were performed on different *E. faecalis* strains according to a previously reported protocol (Kęsik-Szeloch et al., 2013; Yang et al., 2020). The following steps were performed to verify the difference in the adsorption capacity of phage EFap02 to *E. faecalis* EFa02, EFa02R, and EFa02R::*gtr2*. The phage filtrate was diluted to 10^9^ PFU/mL in sterile water to form the initial titer. The host bacteria (1 ml) and phage (10^5^ PFU) were mixed and left in the EP tube for 5 min and centrifuged at 21,000 × g for 2 min. The phage titer was calculated by the DPA method (residual titer). The phage adsorption rate was calculated as [(initial titer −residual titer)/initial titer] × 100%. Three biological replicates were performed.

The phage was diluted in tenfold increments. Then, 100 μl of *E. faecalis* and 4 ml of BHI soft agar were mixed and poured onto a BHI agar plate. Then, 1 μl of the diluted phage was pipetted onto agar plates and incubated at 37°C overnight (Forti et al., 2018).

### Capsule Staining

Bacterial was stained with crystal violet solution for 5 min and then stained with 20% copper sulfate solution. Then, the bacteria were observed under the microscope immediately.

### Biofilm Experiment

Crystal violet staining was used to monitor biofilms (Forti et al., 2018). EFa02, EFa02R, and EFa02R::*gtr2* were inoculated in 1 ml of BHI medium and cultured at 37°C overnight. The OD_600_ was measured, and the culture was diluted to an OD_600_ of 0.02. A diluted bacterial solution (1 ml) was added to a 24-well plate and incubated at 37°C for 24 h, 48 h, or 72 h. The medium was then gently removed, and the wells were washed three times with 1 ml of phosphate-buffered saline (PBS). Finally, the plate was stained with 1 ml of 0.1% crystal violet at room temperature for 20 min. The excess crystal violet was carefully removed, washed with sterile water three times, and air-dried for 3 h. Then, 1 ml of 95% ethanol was added for 10 min, and the OD_570_ of each well was measured.

### Statistical Analysis

Statistical analysis was performed using one-way ANOVA or Student’s *t-*test. A *p* < 0.05 was considered statistically significant.

## Results

### Isolation and Identification of Bacteriophage Against *Enterococcus faecalis*

**Figure 1:**
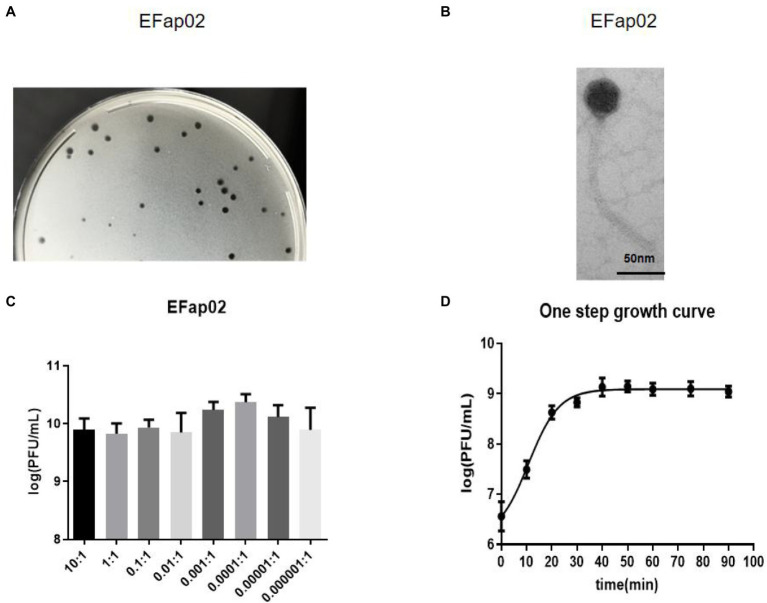
Phage EFap02 general biological characteristics. **(A)** Phage plaques on a double-layer agar plate of EFap02. **(B)** TEM image of phage EFap02. EFap02 has an icosahedral head (length 44 nm ± 5, width 40 nm ± 5) and a long tail (210 nm ± 5). The scale bar in the right corner is 50 nm. **(C)** Different multiplicities of infection (MOI) of EFap02. When the MOI was 0.0001, the EFap02 titer was the highest. **(D)** One-step growth curve of EFap02.

Phage plaques were first discovered on the top agar lawn that contained a clinical strain of *E. faecalis* EFa02. Clear plaques, approximately 2 mm in diameter, were formed on the DLA plate (Figure 1A). The plaque was purified three times, and the isolated phage was named EFap02. Electron microscopy showed that phage EFap02 had an icosahedron head and a long tail of approximately 210 nm (Figure 1B). Phage EFap02 belonged to the Siphoviridae family according to the ICTV guidelines and was named vB_EFaS_EFap02.

### The Bactericidal Effect of Phage EFap02

The highest phage EFap02 amount was 2.3 × 10^10^ PFU/ml when the MOI was 0.0001. Therefore, the optimal MOI of phage EFap02 is 0.0001 (Figure 1C). The one-step growth curve was measured to determine the latent period of phage EFap02 (Figure 1D). The phage titer increased 10 min after infection and peaked at 30 min, indicating a phage lysis time of approximately 30 min. These data suggest that EFap02 is effective at infecting EFa02.

### Stability of Bacteriophage

**Figure 2:**
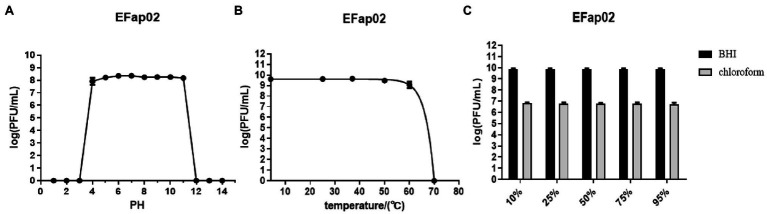
Stability of EFap02 under different conditions. **(A)** EFap02 is stable over a broad range of pH values (pH values from 4 to 11). **(B)** EFap02 is viable over a broad range of temperatures. **(C)** EFap02 is sensitive to chloroform, and the titer drops to approximately 5 × 10^6^ PFU/ml after treatment.

The stability of EFap02 was tested under different pH, temperature, and chloroform treatments. EFap02 was viable when the pH was 4–11 (Figure 2A). Moreover, EFap02 was viable at 60°C and was rapidly inactivated at 70°C (Figure 2B). However, the EFap02 titer dropped to approximately 5 × 10^6^ PFU/mL after chloroform treatment (Figure 2C). These data indicate that EFap02 can tolerate a strong acid and alkali environment and high temperature but is partially sensitive to chloroform.

### Genomic Characterization of Phage EFap02

**Figure 3:**
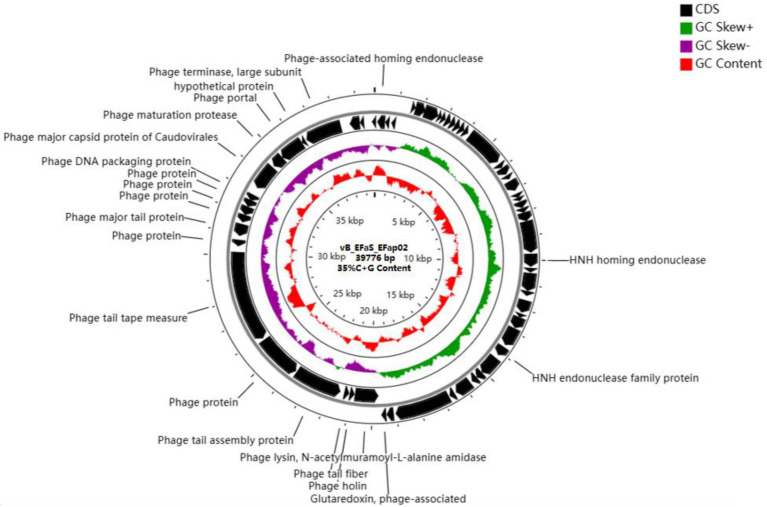
Genome characterization of phage EFap02. Genome map of phage EFap02 and genetic characteristics. Circular genome visualization of EFap02 was performed using the CGView server database. Annotation of the specific function of ORFs was conducted using RAST and the BLASTP database. The first inner circle with the red histogram indicates the GC content, while the second inner circle with the purple and green histograms indicates the GC skew. The outer black circle indicates the predicted ORFs of phage EFap02.

**Figure 4:**
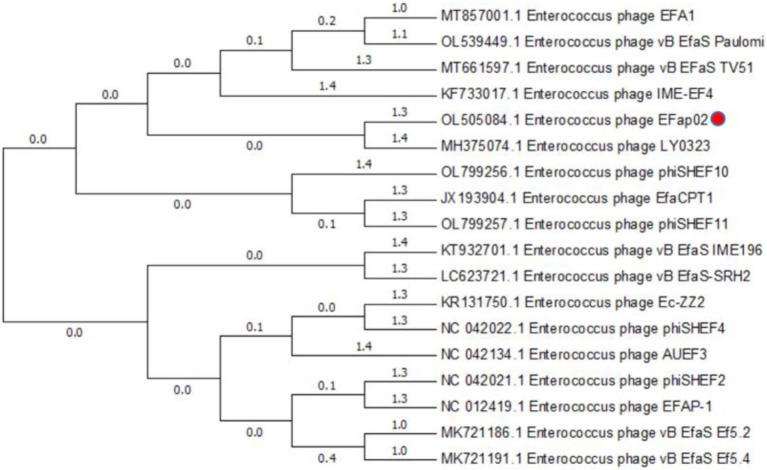
Phylogenetic relationships of phages infecting *Enterococcus faecalis*. Terminase large subunit and the neighbor-joining method were used to construct the phylogenetic tree.

The phage EFap02 genome structure is shown in Figure 3. The whole-genome sequencing results and phageTerm analysis results revealed that EFap02 had a linear double-stranded DNA, with a length of 39,776 bps and a (G + C) content of 35%. The complete genome sequence is available at GenBank (accession no. OL505084). The EFap02 genome sequence was searched as a query in the nucleotide database of the National Center for Biotechnology Information (NCBI). The results showed that the genome sequences of *Enterococcus* phage phiSHEF4 (GenBank accession no. NC_042022.1) and *Enterococcu*s phage phiSHEF11 (GenBank accession no. OL799257.1) shared similar query coverage above 80% and identity above 91% with the EFap02 genome. The phylogenetic tree of EFap02 was constructed based on the nucleotide sequence terminase large subunit (ORF58), as shown in Figure 4. The result showed that EFap02 is closely related to the *Enterococcus* phage LY0323 (GenBank accession no. MH375074) genome, but was distantly related to the other phages included in the analyses. Based on these results, phage EFap02 could be considered as a novel phage. The EFap02 genome sequence encodes 60 predicted open reading frames (ORFs), 20 were functional genes, and 40 were hypothetical proteins (Table 3). No homologs of bacteriophage excisionases, repressors, integrases, or transposases were predicted in the EFap02 genome, supporting phage EFap02 as a lytic phage.

**Table 3:** The ORFs of EFap02.

**ORF**	**Start**	**Stop**	**Strand**	**Function**
ORF01	481	110	−	Phage-associated homing endonuclease
ORF02	681	481	−	hypothetical protein
ORF03	967	764	−	hypothetical protein
ORF04	1,524	1,679	+	hypothetical protein
ORF05	1,676	2095	+	hypothetical protein
ORF06	2061	2,648	+	hypothetical protein
ORF07	2,673	2,792	+	hypothetical protein
ORF08	2,805	2,984	+	hypothetical protein
ORF09	2,996	3,220	+	hypothetical protein
ORF10	3,217	3,429	+	hypothetical protein
ORF11	3,426	3,644	+	hypothetical protein
ORF12	3,641	3,856	+	hypothetical protein
ORF13	3,853	4,050	+	hypothetical protein
ORF14	4,145	5,725	+	hypothetical protein
ORF15	5,817	6,011	+	hypothetical protein
ORF16	6,018	6,338	+	hypothetical protein
ORF17	6,376	6,564	+	hypothetical protein
ORF18	6,637	7,080	+	hypothetical protein
ORF19	7,213	7,398	+	hypothetical protein
ORF20	7,382	7,552	+	hypothetical protein
ORF21	7,566	7,838	+	hypothetical protein
ORF22	7,840	8,046	+	hypothetical protein
ORF23	8,030	8,404	+	hypothetical protein
ORF24	8,397	9,689	+	hypothetical protein
ORF25	9,730	10,269	+	HNH homing endonuclease
ORF26	10,306	10,500	+	hypothetical protein
ORF27	10,514	11,254	+	hypothetical protein
ORF28	11,254	11,463	+	hypothetical protein
ORF29	11,630	12,154	+	hypothetical protein
ORF30	12,211	12,366	+	hypothetical protein
ORF31	12,363	12,842	+	hypothetical protein
ORF32	12,853	13,632	+	hypothetical protein
ORF33	13,726	14,130	+	HNH endonuclease family protein
ORF34	14,277	15,095	+	hypothetical protein
ORF35	15,096	15,386	+	hypothetical protein
ORF36	15,387	15,632	+	hypothetical protein
ORF37	15,713	16,420	+	hypothetical protein
ORF38	16,490	16,714	+	hypothetical protein
ORF39	16,749	19,040	+	hypothetical protein
ORF40	19,107	19,400	+	hypothetical protein
ORF41	19,412	19,618	+	Glutaredoxin, phage-associated
ORF42	20,805	19,708	−	Phage lysin, N-acetylmuramoyl-L-alanine amidase (EC 3.5.1.28)
ORF43	21,041	20,808	−	Phage holin
ORF44	21,301	21,056	−	Phage tail fiber
ORF45	23,711	21,483	−	Phage tail assembly protein
ORF46	25,830	23,755	−	Phage protein
ORF47	30,144	25,912	−	Phage tail tape measure
ORF48	30,712	30,401	−	Phage protein
ORF49	31,451	30,888	−	Phage major tail protein
ORF50	31,895	31,530	−	Phage protein
ORF51	32,299	31,892	−	Phage protein
ORF52	32,631	32,296	−	Phage protein
ORF53	32,902	32,603	−	Phage DNA packaging protein
ORF54	34,453	33,257	−	Phage major capsid protein of Caudovirales
ORF55	35,091	34,528	−	Phage maturation protease
ORF56	36,229	35,078	−	Phage portal (connector) protein
ORF57	36,398	36,234	−	hypothetical protein
ORF58	38,189	36,468	−	Phage terminase, large subunit
ORF59	39,076	38,603	−	hypothetical protein
ORF60	39,277	39,077	−	hypothetical protein

The EFap02 phage genome includes DNA replication and modification, transcription regulation, phage packaging, structural protein, and host lysis protein modules. ORF53 encodes phage DNA packaging protein in the packaging module. ORF58 encodes a large terminase subunit, an enzyme that inserts a single viral genome into the viral procapsid. ORF56, a portal protein, injects DNA into the host cell through a pathway formed by portal protein. Among the structural proteins, ORF49 encodes the phage major tail protein. ORF44 encodes the phage tail fiber. ORF45 encodes the phage tail assembly protein, and ORF47 encodes the phage tail tape measure protein. These structural proteins are involved in binding to the host bacterium. For the lysis module, ORF42 is annotated as N-acetylmuramoyl-L-alanine amidase, and ORF43 is annotated as holin, which can form micron-scale holes in the inner membrane. The phage releases active endolysin (N-acetylmuramoyl-L-alanine amidase) into the periplasm to digest the peptidoglycan of *E. faecalis*. The Virulence Search Database shows that none of the ORFs encode virulence factors or antibiotic resistance genes, indicating that EFap02 can be safely used to treat *E. faecalis*-associated diseases.

### Isolation and Sequencing of the Phage-Resistant Strain EFa02R

**Figure 5:**
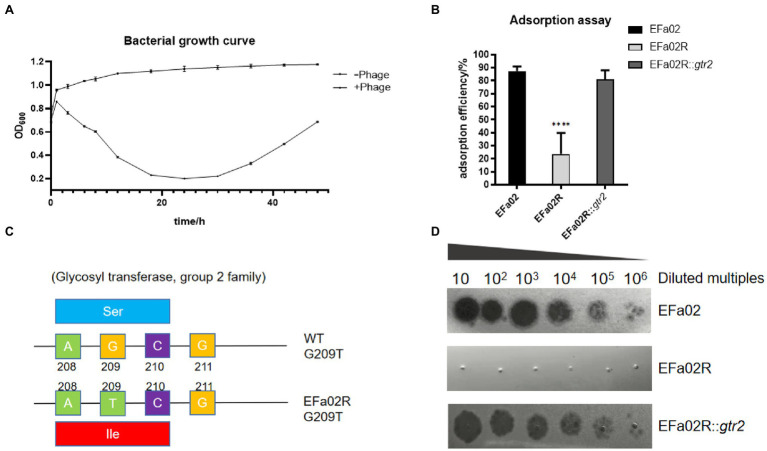
**(A)** Growth curve of the phage–bacteria coculture. Thirty hours after phage addition, the OD_600_ of the phage and bacteria coculture began to increase. **(B)** Adsorption rate of phage onto three strains. This result indicates that EFap02 can adsorb EFa02 and EFa02R::*gtr2* but not EFa02R (**** *p* < 0.0001). **(C)** The mutation site of the glycosyltransferase Group 2 gene in EFa02R; the mutant site is G209T, which results in the amino acid change from serine to isoleucine. **(D)** EOP assay indicates that EFap02 can infect EFa02 and EFa02R::*gtr2* but not EFa02R.

In the bacterial growth curve, the OD_600_ of the phage–bacteria coculture increased 30 h after the phage was added (Figure 5A). The culture medium became turbid due to the growth of phage-resistant bacteria. We then isolated a phage-resistant mutant named EFa02R. Since most resistant bacteria exhibit phage resistance mediated by receptor mutation (Labrie et al., 2010), an adsorption assay was conducted. The results showed that the adsorption rate of EFa02R decreased to approximately 20% (Figure 5B). After sequencing, it was found that resistant bacteria contain a mutation in the *gtr2* gene. The mutant site changed from AGC to ATC, and the amino acid changed from serine to isoleucine (Figure 5C).

### Identification of the Phage EFap02 Receptor

**Figure 6:**
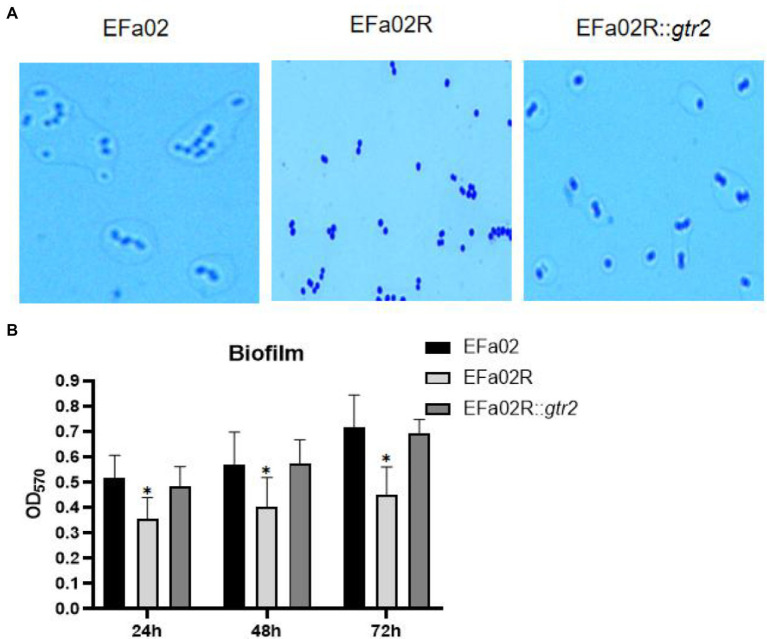
**(A)** Capsule staining of EFa02, EFa02R, and EFa02R::*gtr2.* Capsule staining showed that EFa02 and EFa02R::*gtr2* have the capsule, but EFa02R lost the capsule. **(B)** The biofilm mass of EFa02R was less than those of EFa02 and EFa02R::*gtr2* (*p < 0.05) at the given time points.

The *gtr2* gene is related to the synthesis of capsular polysaccharide on the surface of *Acinetobacter baumannii* (Kenyon and Hall, 2013; Singh et al., 2018). We also performed capsule staining of EFa02, EFa02R and EFa02R::*gtr2*, which showed that EFa02 and EFa02R::*gtr2* have the capsule, but EFa02R lost the capsule (Figure 6A). Furthermore, we performed a phage adsorption experiment to test whether capsular polysaccharide is the receptor for EFap02. We found that the adsorption capacity of EFa02R dropped dramatically to 23.9%, and the adsorption capacity recovered to 89% after complementing the *gtr2* gene (Figure 5B). Additionally, EOP testing showed that EFa02R::*gtr2* was resensitive to EFap02 (Figure 5D). These experiments suggest that the mutation of *gtr2* leads to the loss of capsular polysaccharides and prevents EFap02 from adsorbing to EFa02R.

### EFa02R Is Impaired in Biofilm Formation

We measured the biofilm formation of these strains on the polystyrene surface after 24, 48, and 72 h of incubation. At each time point, the biofilm of the resistant mutant EFa02R was much less than that of the wild-type strain EFa02 and the complemented strain EFa02R::*gtr2* (Figure 6B), indicating that the phage-resistant mutant had impaired biofilm formation.

## Discussion

Phage therapy has been used to treat *E. faecalis* infectious diseases (Letkiewicz et al., 2009; Bolocan et al., 2019), as well as many other diseases. For example, phages can treat alcoholic hepatitis by killing the *E. faecalis* that produces cytolysin which leads to hepatocyte lysis (Duan et al., 2019). Thus, the characterization of *E. faecalis* phages is important for treating *E. faecalis-associated* diseases.

Identification of the phage receptors is essential for the rational selection of phages for therapy. The *E. faecalis* phage receptors had been descried previously. Duerkop et al. found that phage infection of *E. faecalis* requires a predicted integral membrane protein named PIP_EF_ (phage infection protein of *E. faecalis*). PIP_EF_ is an integral membrane protein that spans the membrane six times (Duerkop et al., 2016). In 2018, Ho et al. showed that mutation of the glycosyltransferase *epaR* leads to the resistance to phage NPV1 by preventing phage adsorption (Ho et al., 2018). And these phage receptors are cell wall-associated structures that are important for cell viability under external stresses (Canfield and Duerkop, 2020).

In this study, the isolated EFap02 phage is stable under high temperatures and other conditions, making it convenient to store and transport for future clinical use. Although EFap02 has strong lytic activity, we observed the rapid emergence of phage-resistant mutants. Resistance is an issue in phage therapy, and its mechanisms are diverse. The receptor mutation is the most likely to occur, and it prevents phage adsorption (Labrie et al., 2010). In this study, the phage-resistant strain EFa02R had loss-of-function mutations in the glycosyltransferase gene *Group 2 family* responsible for the biosynthesis of capsular polysaccharides (Kenyon and Hall, 2013; Singh et al., 2018). We confirmed that the *gtr2* mutation in EFa02 causes phage resistance and that the capsular polysaccharide is the phage receptor. Identifying phage receptors is essential for the rational development of phage cocktails (Gordillo Altamirano and Barr, 2021).

The loss of receptors prevents phage adsorption; however, there is always a trade-off for phage resistance. We observed that the capsular polysaccharide-loss phage-resistant mutant reduced the formation of biofilms. Previous studies found that the formation of biofilms increased the resistance to antibiotics (Ch'ng et al., 2019). Capsular polysaccharide loss may make bacteria resensitive to some antibiotics, which may allow for clearance of the bacteria by the body’s immune system or antibiotics (Gordillo Altamirano and Barr, 2021).

In summary, this study revealed the biological and genomic characteristics of a phage EFap02 and identified its receptor as the capsular polysaccharide. This study suggests EFap02 as a potential phage therapy agent.

## Data Availability Statement

The datasets presented in this study can be found in online repositories. The names of the repository/repositories and accession number(s) can be found at: https://www.ncbi.nlm.nih.gov/genbank/, OL505084.

## Author Contributions

JL, QZ, KX, and JW conceived and designed the experiments. JL performed the experiments. JL, YZ, YLi, and YLu analyzed the data. JL, KX, QZ, and JW wrote the paper. All authors contributed to the article and approved the submitted version.

## Funding

This research was supported by the National Natural Science Foundation of China (NSFC, 32000120 to QZ) and Military Youth Training Program (2019XQN14).

## Conflict of Interest

The authors declare that the research was conducted in the absence of any commercial or financial relationships that could be construed as a potential conflict of interest.

## Publisher’s Note

All claims expressed in this article are solely those of the authors and do not necessarily represent those of their affiliated organizations, or those of the publisher, the editors and the reviewers. Any product that may be evaluated in this article, or claim that may be made by its manufacturer, is not guaranteed or endorsed by the publisher.

## Footnotes

^1^ https://rast.nmpdr.org/

^2^ http://linux1.softberry.com/

^3^ https://blast.ncbi.nlm.nih.gov/Blast.cgi

^4^ https://galaxy.pasteur.fr

^5^ http://www.megasoftware.net
